# Expression of HES and HEY genes in infantile hemangiomas

**DOI:** 10.1186/2045-824X-3-19

**Published:** 2011-08-11

**Authors:** Omotinuwe Adepoju, Alvin Wong, Alex Kitajewski, Karen Tong, Elisa Boscolo, Joyce Bischoff, Jan Kitajewski, June K Wu

**Affiliations:** 1 Department of Surgery, College of Physicians and Surgeons, 630 W168th St, New York, 10032, USA; 2 Departments of Obstetrics & Gynecology & Pathology, College of Physicians and Surgeons, 630 W168th St, New York, 10032, USA; 3 Vascular Biology Program and Department of Surgery, Children's Hospital Boston and Harvard Medical School, 300 Longwood Avenue, Boston, 02115, USA

**Keywords:** hemangioma, hemangioma stem cells, Notch receptors, Notch signaling, HES/HEY transcription factors

## Abstract

**Background:**

Infantile hemangiomas (IHs) are the most common benign tumor of infancy, yet their pathogenesis is poorly understood. IHs are believed to originate from a progenitor cell, the hemangioma stem cell (HemSC). Recent studies by our group showed that NOTCH proteins and NOTCH ligands are expressed in hemangiomas, indicating Notch signaling may be active in IHs. We sought to investigate downstream activation of Notch signaling in hemangioma cells by evaluating the expression of the basic HLH family proteins, HES/HEY, in IHs.

**Materials and Methods:**

HemSCs and hemangioma endothelial cells (HemECs) are isolated from freshly resected hemangioma specimens. Quantitative RT-PCR was performed to probe for relative gene transcript levels (normalized to beta-actin). Immunofluorescence was performed to evaluate protein expression. Co-localization studies were performed with CD31 (endothelial cells) and NOTCH3 (peri-vascular, non-endothelial cells). HemSCs were treated with the gamma secretase inhibitor (GSI) Compound E, and gene transcript levels were quantified with real-time PCR.

**Results:**

*HEY1*, *HEYL*, and *HES1 *are highly expressed in HemSCs, while *HEY2 *is highly expressed in HemECs. Protein expression evaluation by immunofluorescence confirms that HEY2 is expressed by HemECs (CD31+ cells), while HEY1, HEYL, and HES1 are more widely expressed and mostly expressed by perivascular cells of hemangiomas. Inhibition of Notch signaling by addition of GSI resulted in down-regulation of *HES/HEY *genes.

**Conclusions:**

*HES/HEY *genes are expressed in IHs in cell type specific patterns; HEY2 is expressed in HemECs and HEY1, HEYL, HES1 are expressed in HemSCs. This pattern suggests that *HEY/HES *genes act downstream of Notch receptors that function in distinct cell types of IHs. *HES/HEY *gene transcripts are decreased with the addition of a gamma-secretase inhibitor, Compound E, demonstrating that Notch signaling is active in infantile hemangioma cells.

## Background

Infantile hemangiomas (IHs) are the most common benign tumors of infancy. Despite their prevalence, the pathogenesis of IHs is not well understood. IHs are characterized by three phases: proliferating, involuting and involuted phases. These are defined by a period of rapid proliferation of blood vessels in the first year of life, followed by gradual regression of the vascular component with replacement by fibro-fatty tissue.[1,2]

The Notch family of proteins function as cell surface receptors, is highly conserved over multiple species, and is involved in cell fate determination during embryogenesis.[3] Notch genes function critically in angiogenesis and arteriogenesis.[4-8] The Notch family of genes consists of four Notch receptors (Notch1, -2, -3, and -4),[9-13] as well as two classes of ligands, Delta-like (Dll-1, -3, -4),[14-16] and Jagged (Jagged-1, -2)[17,18].

Previous work in our laboratory has shown that members of the Notch signaling pathway, namely receptors and ligands, are expressed in hemangiomas.[19] In particular, we have demonstrated that the endothelial-associated *NOTCH1*, *NOTCH4*, and *JAGGED-1 *genes are highly expressed in involuting hemangiomas and HemECs, while *DELTA-LIKE LIGAND-4 (Dll4) *showed an intermediate level of expression in HemECs. In contrast, *NOTCH3*, normally expressed in vascular smooth muscle cells, was found expressed in HemSCs. Moreover, studies by immunofluorescence showed that NOTCH1 and NOTCH4 were expressed in HemECs, whereas NOTCH3 was present in perivascular cells in IH tissue.[20]

The purpose of this study is to evaluate the expression of downstream effector genes of Notch signaling, specifically the HES and HEY family of transcription factors. *HES/HEY *genes are part of the hairy enhancer of split related (HESR) family of genes and encode basic helix-loop-helix proteins that function as transcriptional repressors. After activation by binding to its ligand, the transmembrane Notch receptor undergoes cleavage and release of the intracellular domain (NICD). This NICD then translocates to the nucleus, where it interacts with the DNA-binding protein RBPJκ. NICD interaction with RBPJκ leads to transcription of Notch target genes. The primary target genes of Notch signaling are the HES/HEY family of transcriptional regulators. [21]

We asked whether the expression of *HES/HEY *transcripts and proteins are found in cell types associated with IHs, as further evidence for a role for Notch receptor signaling in IHs.

## Methods

### Preparation of hemangioma specimen

IRB approval for collection of resected hemangiomas was obtained from Columbia University College of Physicians & Surgeons (IRB #AAAA9976). Tissues were either paraffin embedded for sectioning or used immediately for cell isolation for *in vitro *experiments.

### Cell extraction and isolation

**Figure 1:**
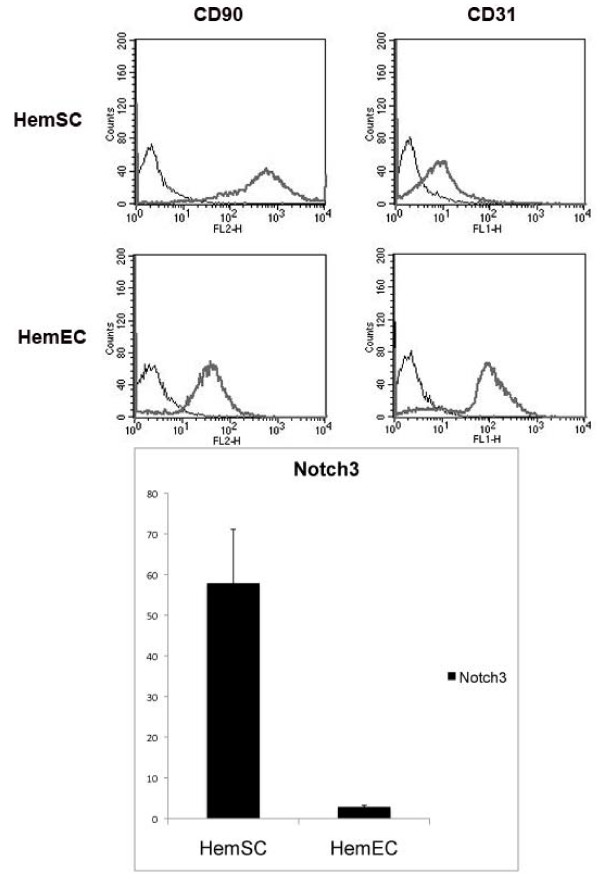
**FACS analysis**. (a) FACS analysis results show that HemSCs are positive for CD90 and negative for CD31, consistent with previously published reports,[22] whereas HemECs are positive for CD31. ***NOTCH3 *expression**. (b) qPCR analysis showed that HemSCs express high levels of *NOTCH3*, while HemECs showed minimal to no NOTCH3 expression.

Previous published reports showed that HemSCs expressed CD90 but did not express CD31.[22]. We characterized HemSCs based upon CD90 expression and lack of CD31 expression and verified that the isolated HemSCs expressed high levels of *NOTCH3*, relative to mesenchymal stem cells. [20] (MSC, commercially available from Lonza, Figure 1). HemECs were confirmed by lack of Notch3 transcripts by qPCR and CD31 positivity by FACS analysis.

HemSC and HemEC isolation was conducted as previously described.[22] Briefly, the hemangioma samples were minced into small pieces with a scalpel and digested using collagenase (Roche). From this single cell suspension, cells expressing CD133 were selected using a magnetic microbead cell sorting system (Miltenyi Biotec), and plated on fibronectin-coated plates in EGM-2 media (Lonza) supplemented with 20% fetal bovine serum (Invitrogen), penicillin, and streptomycin (Invitrogen). CD133 negative cells were plated in the same manner. These cells were later sorted for endothelial cells using CD31 antibody-coated magnetic Dynal beads (Invitrogen). RNA was later isolated from both cell populations (CD133+, or HemSC, and CD31+, or HemEC) as described below. Cells were isolated from 3 different patients (H37, H40, and H41). Furthermore, RNA from isolated, characterized and validated HemSC and HemEC samples[22] was provided by EB and JB.

### RNA isolation and RT-PCR

Total RNA isolation was performed with RNeasy Mini Prep Kit (Qiagen). Quantity of RNA was determined with spectrophotometry. cDNA was synthesized by reverse transcription of 1 microgram of total RNA, performed using the Superscript II system (Invitrogen). All reverse transcription (RT) reactions were amplified by PCR to determine expression of beta-actin, to confirm successful generation of cDNA.

### PCR

Primers used were for *HEY1*, *HEY2*, and *HEYL*, and *HES1, HES3, HES5, HES6, and HES7*. *HEY1*, forward: 5' ACG AGA ATG GAA ACT TGA GTT C 3', reverse: 5' AAC TCC GAT AGT CCA TAG CAA G 3'. *HEY2*, forward: 5' ATG AGC ATA GGA TTC CGA GAG TG 3', reverse: 5' GGC AGG AGG CAC TTC TGA AG 3'. *HEYL*, forward: 5' CAG GAT TCT TTG ATG CCC GAG 3', reverse: 5' GAC AGG GC5T GGG CAC TCT TC 3'. *HES1*, forward: 5' CCC AAC GCA GTG TCA CCT TC 3', reverse: 5' TAC AAA GGC GCA ATC CAA TAT G 3'. *HES3*, forward: 5' CAT CAA TGT GTC ACT GGA GCA G 3', reverse: 5' CAA GGA GTT CTG AAG GCT TCT C 3'. *HES5*, forward: 5' TCG CCT CGT CGC CTG TTC 3', reverse: 5' CCA CGA GTA GCC TTC GCT GTA G 3'. *HES6*, forward: 5' AGA ACG CCG AAG TGC TGG AG 3', reverse: 5' GAA CTG AGT CAG GCT CCT GCT G 3'. *HES7*, forward: 5' GGA ACC CGA AGC TGG AGA AAG 3', reverse: 5' GCC TCG GAT CTA CCG GCT TG 3'.

Survey PCR was performed as follows with the Eppendorf Mastercycler: initiation with heating to 94°C for 2 minutes followed by 95°C for 45 seconds. The program then proceeded to cycle at 60°C (1 minute), annealing at 72°C (1 minute) and extension 72°C (5 minutes) for 40 cycles. Mesenchymal stem cells (MSC, Lonza) were used as controls.

### Quantitative Real-time PCR

Real time PCR was performed in triplicate utilizing the Applied Biosystem 7300 and SYBR Green PCR Master Mix reagent (Applied Biosystems). Primers used were described in the above section.

The PCR cycler was programmed as follows: initiation with heating to 50°C for 2 minutes followed by 95°C (2 minutes). The program then proceeded to cycle at 95°C (15 seconds), annealing at 60°C (40 seconds) and extension at 72°C (30 seconds) for 50 cycles. The results were normalized to beta-actin levels and the triplicate results were averaged for each sample.

DNA from human dermal microvascular endothelial cells (HDMEC) (isolated from neonatal foreskin as described for HemECs) and MSCs were used as controls.

### Immunofluorescence

Paraffin-embedded hemangioma specimens were stained for NOTCH3, (Abcam 23426, rabbit polyclonal, 1:75), HEY2 (Millipore AB5716, rabbit polyclonal, 1:100) and HEYL (Millipore MAB10094, mouse monoclonal, 1:250). The specimens were also co-stained for Notch3 and HEYL, CD31 (DAKO M0823, mouse monoclonal, 1:20) and HEY1 (Millipore AB5714, rabbit polyclonal, 1:100), and CD31 and HES1 (Santa Cruz SC25392, rabbit polyclonal, 1:50), and visualized by immunofluorescence. Briefly, the slides were de-paraffinized and incubated in blocking solution containing avidin, biotin, and animal serum. The blocking agent was removed and the sections were incubated in primary antibody overnight at 4 degrees. The slides were washed and incubated with secondary antibody at room temperature for 30 minutes. Slides were then incubated with immunofluorescent dye (Alexa green 488 or red 594) and Vectashield with DAPI (Vectorlabs) was applied for visualization of nuclei.

### GSI treatment of Hemangioma Stem Cells

HemSCs were grown to confluence in EGM-2 (Lonza EBM-2 with Bullet Kit) supplemented with 20% FBS. 1.5-2 × 10^6 ^cells were plated in a 10 cm plate and left overnight at 37°C or until at least 80% confluent. Either 500 nM of compound E (KRICT, South Korea) in 4 microliters, or 4 microliters of vehicle (DMSO) was added to the plate. RNA was harvested from the control and treated cells 24 hours later and used for transcript analysis as described above. Compound E manufactured by KRICT is equivalent to Compound E from Merck.

### Statistical Analysis

For qPCR results, gene expression levels were measured in triplicate and normalized to beta-actin for an assigned relative value and standard deviation. Statistical significance between 2 samples was calculated using the unpaired Student's *t-test*.

## Results

### Hemangioma stem cell and endothelial cell expression of HES and HEY genes

We surveyed HemSC and HemEC samples by RT-PCR to determine which of the *HES/HEY *genes are expressed, including use of HemSC/EC 131 and HemSC/EC 133 (characterized and provided by EB and JB).[22] While *HES1, -5*, and *-7 *have been shown to be induced by Notch signaling, *HES2, -3*, and *-6 *are thought to be Notch independent.[21] Since little is known about *HES4 *expression patterns,[23] and no published, validated unique primer sets are available for *HES2 and HES4*, we chose not to evaluate *HES2 *and *HES4*.

**Figure 2:**
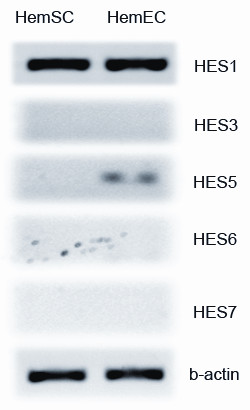
**Survey PCR for *HES *gene**. PCR analysis showed that HemSCs showed no expression of *HES3, -5, -6, or 7*; a very low level of expression was seen for *HES5 *in HemECs.

Our initial survey analysis of expression of *HES/HEY *genes in HemSCs and HemECs thus included evaluation of *HES1*, *HES3*, *HES5*, *HES6*, *HES7*, *HEY1*, *HEY2*, *HEYL*. We found that *HEY1*, *HEY2*, and *HEYL *were all expressed, in HemSCs and HemECs. Of the HES genes surveyed, only *HES1 *was expressed, whereas *HES3*, *HES5, HES6, and HES7 *were not (Figure 2). As a result, further analyses were performed for the *HEY *genes and *HES1 *only.

**Figure 3:**
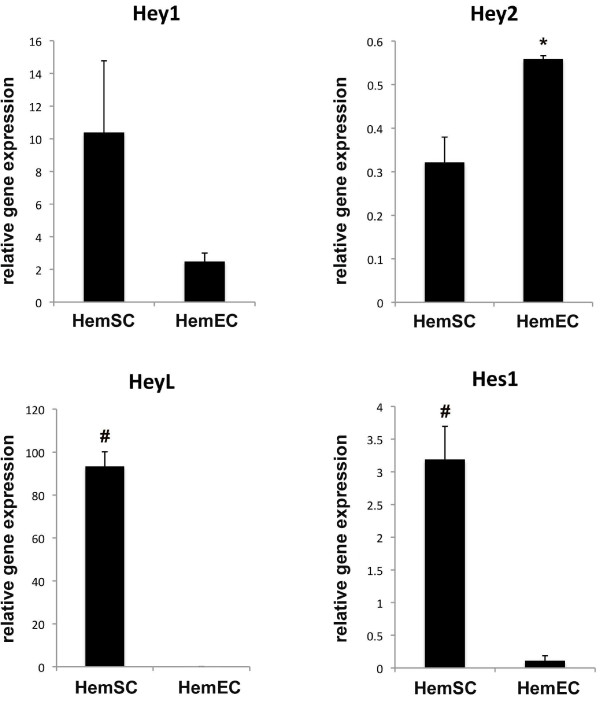
**Quantitative PCR results of *HES *and *HEY *genes in HemSCs and HemECs**. Quantitative PCR results showed that *HES1*, *HEY1*, and *HEYL *had higher transcript levels in HemSCs when compared to HemECs (*p<0.05, #p<0.01.) Gene transcript levels were expressed as relative to beta-actin levels. Results were done in triplicates at least in 2 separate experiments and representative of 2 different cell lines. Statistical analysis showed that the difference in transcript levels for *HEY1 *approached, but did not achieve, statistical significance (p = 0.08). However, the transcript levels were statistically different between HemSCs and HemECs: Hey2 (p = 0.02), HeyL (p = 0.002), and Hes1 (p = 0.008).

We asked whether the expression of *HEY1*, *HEY2*, *HEYL *and *HES1 *genes was found in distinct cell types of IHs; HemSCs and HemECs. We found that HemSCs had high relative transcript levels of *HEYL *and *HES1*, when compared to the expression of these genes in HemECs. In contrast, HemECs expressed *HEY2 *at higher levels than HemSCs. The relative transcript levels of *HEY1 *between HemSCs and HemECs were not statistically significant (Figure 3).

We used immunoflurescence to evaluate the expression of HES and HEY proteins in IHs and to confirm the expression analysis conducted on transcripts levels in cells derived from IHs. Immunofluorescence studies showed that HES1, HEY1, HEY2, HEYL were expressed in IHs (data not shown). In order to further clarify the staining patterns of these proteins, IH samples were co-stained with antibodies for HES or HEY and either CD31, an endothelial cell marker, or NOTCH3, which is present in non-endothelial peri-vascular cells [20].

**Figure 4:**
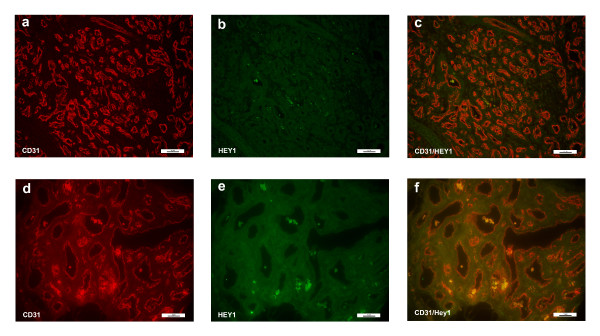
**Immunofluorescence staining of CD31 and HEY1**. Immunofluorescence staining CD31 (red) and HEY1 (green). There was no appreciable staining in the involuting hemangioma specimen (bottom panels, d-f). However, HEY1 was present in the proliferating hemangioma (a-c). Magnification, 40X.

Co-staining studies showed that HEY1 was weakly expressed in cells of both proliferating and involuting hemangiomas. The HEY1 staining was low and diffuse throughout the cell. The low level of HEY1 staining did not co-localize with endothelial cells (Figure 4), and thus was not deemed to be endothelial-specific, in contrast to normal expression of hey1 in murine vasculature.[24,25]

**Figure 5:**
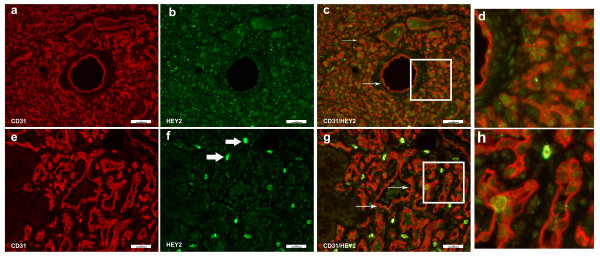
**Immunofluorescence staining of CD31 and HEY2**. Immunofluorescence staining of CD31 (red) and HEY2 (green). Proliferating hemangioma, panels a-c; involuting hemangioma, panels e-g. HEY2 staining was seen in the endothelial cell nucleui, whereas CD31 staining was present in the cytoplasm. Therefore, while CD31 and HEY2 co-localized, the color did not overlap (thin arrows, panels c & g). Not all endothelial cells (red cytoplasmic staining) co-stained with HEY2, and there were non-endothelial cells that were HEY2 positive (Figure 5f, thick arrows, bright green). Magnification, 40X. Panels d, h: close up of panels 5c (proliferating hemangioma) and 5g (involuting hemangioma).

Evaluation of HEY2 proteins levels by IF showed high expression in proliferating IHs with predominantly nuclear localization (Figure 5b). Involuting IHs also showed HEY2 expression (Figure 5f), albeit with less intensity of staining as that seen for proliferating IHs, with the exception of sporadic stromal cells that stained very strongly for HEY2 in involuting IHs (Figure 5f, thick arrows). Although HEY2 was expressed in several different cell types (Figure 5b, f), HEY2 proteins were often found to be expressed in cells that were positive for the endothelial cell marker CD31 (Figure 5c, g) and one can clearly see the nuclear HEY2 in cells that show CD31 cytoplasmic staining. HEY2 was not uniformly present in all endothelial cells (Figure 5c, g, arrows), suggesting that only a subset of IH endothelial cells have Notch target, HEY2 expressed.

**Figure 6:**
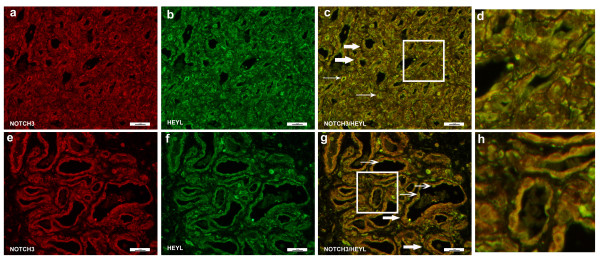
**Immunofluorescence staining of NOTCH3 and HEYL**. Immunofluorescence staining of NOTCH3 (green) and HEYL (red). Proliferating hemangioma, panels a-c; involuting hemangioma, panels e-g. NOTCH3+ cells are located in the perivascular regions and co-localize with HEYL (thick arrows, panels c & g). However, some endothelial cells also express HEYL (thin arrows, panels c & g). Magnification, 40×. Panels d, h: close up of Figure 6c (proliferating hemangioma) and 6g (involuting hemangioma). There were luminal cells that stained for HEYL (green, thick arrows) but not NOTCH3. The majority of HEYL positive cells co-localized with NOTCH3 (yellow).

Evaluation of HEYL (Figure 6) protein levels showed expression in multiple cell types but strong co-localization of expression with NOTCH3 positive cells in both proliferating (Figure 6c) and involuting (Figure 6g) IHs. We have previously established NOTCH3 expression is high in perivascular cells, CD133 positive components of IHs (HemSCs and/or perivascular cells) and in isolated HemSCs.[20] A subset of cells expressed HEYL but not NOTCH3, shown as luminal cells with green staining (HEYL) (Figure 6c, g). We thus conclude that HEYL is predominantly expressed in NOTCH3 positive cells of IHs.

**Figure 7:**
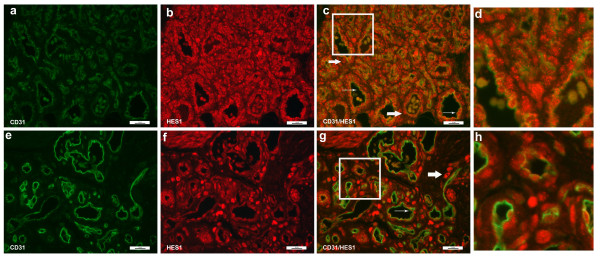
**Immunofluorescence staining of CD31 and HES1**. Immunofluorescence staining of CD31 (green) and HES1 (red). Proliferating hemangioma, panels a-c; involuting hemangioma, panels e-g. HES1 was expressed in HemSCs and were localized in the peri-vascular cells (thick arrows, panels c & g). There were occasional endothelial cells that showed nuclear staining of HES1 and cytoplasmic staining of CD31 (thin arrows, panels d & f). Magnification, 40×. Panels d, h: close up of Figure 7c (proliferating hemangioma) and 7g (involuting hemangioma). There were occasional endothelial cells that expressed HES1 (red nuclei with green cytoplasm, thin arrows), while the majority of HES1+ cells (red nuclei) were located in non-endothelial perivascular cells.

Evaluation of HES1 expression showed strong nuclear stain in a variety of cells in both proliferating and involuting IHs (Figure 7). The HES1 staining was predominantly nuclear. A very low percentage of CD31+ endothelial cells co-stained for HES1 (Figure 7c, g, thin arrows), indicating rare expression of HES1 in the endothelial component of IH. In contrast, HES1 was strongly expressed on perivascular cells, possibly pericytes and HemSCs (thick arrows). This was clearly evident in involuting IHs, as strong nuclear signal was seen on a subset of perivascular cells and cells within the stromal compartment.

### *HES/HEY *genes are activated by Notch signaling

**Figure 8:**
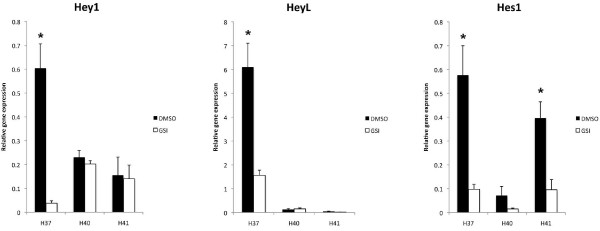
**Quantitative PCR results of HES/HEY genes after treatment with gamma secretase inhibitor**. Transcript levels of *HES*/*HEY *genes in HemSCs are changed with administration of a gamma secretase inhibitor (GSI), Compound E. *HEY1 *(left) transcripts were universally decreased after GSI treatment, although only statistically significant for H37 (p = 0.01). *HEYL *(middle) transcripts were decreased in H37, (p = 0.02), but baseline transcript levels were too low for the other 2 cell lines for meaningful comparison. *HES1 *(right) transcripts were universally decreased and were statistically significant for H37 (p = 0.02) and H41 (p = 0.03). It was not significant for H40 (p = 0.1).

Notch signaling is dependent on gamma-secretase cleavage to induce transcriptional activation of CSL and thus drive Notch target gene expression, such as Hes and Hey genes.[26] When HemSCs isolated from IHs were treated with a Compound E, a gamma-secretase inhibitor, the transcript levels of several HES/HEY genes were reduced, indicating active Notch signaling maintained their expression (Figure 8). In HemSCs from the H37 sample, all the tested *HES/HEY *genes (*HEY1, HEYL, HES1*) transcript levels were downregulated by GSI, with statistical significance (*HEY1*, p = 0.01; *HEYL *and *HES1*, p = 0.02). In sample H40 and H41, HemSCs showed variable changes in *HES/HEY *transcript levels (Figure 8), possibly due to low baseline *HES/HEY *transcript levels, compared to H37 HemSCs at baseline (dark columns). Transcript levels of HEY2 also were low at baseline (10% of that of *HEY1 *and *HES1*, and 1% of that for *HEYL*) and were difficult to evaluate after GSI treatment (data not shown).

Taken together, these results demonstrated that the Notch receptor is active and induces transcription of downstream genes in HemSC. A subset of *HES/HEY *genes responded to GSI treatment, and suppression of Notch signaling activity reduced the transcript levels of these genes. *HES1 *transcript levels were consistently down-regulated by GSI, and *HEY1 *was variably down-regulated. H37 sample, which had higher baseline *HES/HEY *transcript levels than cells from other hemangioma specimens, showed consistent and dramatic down-regulation of *HES/HEY *expression in HemSCs.

## Discussion

Notch proteins function as receptors on the cell surface and are activated via interaction with Notch ligands, which are also located on the cell surface. This juxtacrine signaling leads to proteolytic cleavage of the Notch ICD, catalyzed by the γ-secretase complex. The intracellular domain of Notch (NICD) is released and translocates into the cell nucleus, where it binds with the CSL complex, leading to activation of target genes such as the Hes/Hey family of transcription factors.[27-29] As such, expression of Hes and Hey genes is used to indicate that Notch signaling is functional in a cell. Our previous study showed that Notch receptors and ligands were expressed in HemSCs and HemECs and we were interested in determining if Notch target genes were expressed in these cells.

After analysis of eight members of the HES/HEY family, we found that HES1, HEY1, HEY2 and HEYL are expressed in infantile hemangiomas. Moreover, similar to Notch receptors and ligands, there are differences in transcript levels of these genes in the different cell types. This may suggest specificity in Notch receptor downstream signaling. Alternately, this specificity may reflect that distinct HES/HEY genes are inherent to different cell types. Moreover, our immunofluorescence results also parallel the Notch staining patterns previously reported, specifically, that endothelial cells and perivascular cells stain for different Notch receptors.[20] HES1 and HEYL were predominantly present on perivascular cells, however, occasional endothelial cells also expressed these transcription factors. HEY2, an endothelial-associated protein, mostly co-localized to endothelial cells (CD31+) in IHs, however there were occasional non-endothelial, perivascular cells that showed HEY2 staining. Nonetheless, their presence suggests that Notch signaling is active in these cells. Thus we conclude that HES1 and HEYL are expressed in perivascular cells of IHs, whereas HEY2 is specific to the endothelial component of IHs.

Gamma secretase inhibitors (GSI) block proteolytic cleavage by γ-secretase and thus prevent the release of the NICD into the nucleus and subsequent downstream steps. Notch functions are inhibited or attenuated by GSI treatment, and it has been established that GSI treatment can elicit angiogenic phenotypes associated with loss of Notch function [30,31]. GSI treatment has also been used to evaluate Notch function during wound healing[32,33]. Moreover, HES1 transcripts in melanoblasts[34] and HEY2 transcripts in human mesenchymal stem cells[7] have been shown to be decreased with gamma secretase inhibitor treatment. However, it was not clear whether they also tested other *HES/HEY *genes. As expected, treatment of our cells with the GSI Compound E showed a decrease of *HES/HEY *gene transcription. This suggests that not only are Notch receptors and ligands expressed in HemSCs [20], but that Notch signaling is occurring in HemSCs.

Since there was variability in transcript levels of the different *HES/HEY *family proteins when HemSCs and HemECs were compared, there may be target specificity of *HES/HEY *genes in the Notch signaling pathway. In HemSCs, where NOTCH3 is strongly expressed, HES1, HEY1, and HEYL were expressed at levels 10 to 100 times to that of HEY2. It is conceivable that NOTCH3 specifically induces transcription of HES1, HEY1, and HEYL, whereas transcription of HEY2 is activated by other Notch receptors.

Measurement of these effector genes will provide a tool for exploring Notch functions and relating that expression to the patholobiology of IHs. Moreover, identification of transcription factors critical to hemangioma development may provide multiple specific therapeutic targets along the Notch signaling axis. In a recent study by Boscolo et al, JAGGED-1 was found to be critical in a stem-cell to pericyte differentiation in a murine model of hemangioma.[35] Since JAGGED-1 exerts its activities by signaling through Notch receptors, its crucial role in regulating HemSC differentiation to pericytes suggests that the Notch pathway plays a role in hemangioma pathophysiology. Future studies will concentrate on the effects of functional manipulation of Notch signaling on cell behavior, and more specific inhibition of specific Notch receptors.

## Conclusion

In summary, we surveyed HEY1, HEY2, HEYL, and HES1, HES3, HES5, HES6, and HES7 expression in infantile hemangiomas in this study. We showed that HES1, HEY1, and HEYL are highly expressed in HemSCs, whereas HEY2 is expressed in HemECs. Moreover, *HES *and *HEY *transcripts are affected by Compound E, a gamma secretase with consistent down-regulation of HES1, showing that NOTCH signaling occurs in infantile hemangiomas.

## Competing interests

The authors declare that they have no competing interests.

## Authors' contributions

OA performed all the immunofluorescence and design of the experiments and some qPCR experiments, prepared manusript. AW performed all the Compound E inhibition experiments, some qPCRs, and some immunofluorescence, interpretation of data. AK and KT performed qPCRs and cell culture maintenance. EB and JB isolated and provided some of the cells for experiments. JK supervised the conceptual design and implementation of the experiments, interpretation of data, and preparation of manuscript. JKW isolated some of the cells, performed some qPCRs and prepared the manuscript. All authors have read and approved the final version.
